# Weekend Warrior and Other Leisure-Time Physical Activity Patterns in Relation to Positive Self-Rated Health: Racial Differences Among Brazilian University Students

**DOI:** 10.3390/ijerph23050599

**Published:** 2026-05-01

**Authors:** Thiago Ferreira de Sousa, Karine Moraes Pereira, Ysamara dos Santos Conceição, Cristiane dos Santos Matos, Djalma Pereira Santana, Aline de Jesus Santos, Chandra Lima Maciel, Grasiely Faccin Borges

**Affiliations:** 1 Department of Health Sciences, State University of Santa Cruz, Ilhéus 45662-900, Brazil; kmpereira.ppgef@uesc.br (K.M.P.); ysconceicao.ppgef@uesc.br (Y.d.S.C.); csmatos.ppgef@uesc.br (C.d.S.M.); dpsantana.ppgef@uesc.br (D.P.S.); clmaciel@uesc.br (C.L.M.); gfborges@uesc.br (G.F.B.); 2 Department of Education, State University of Bahia, Teixeira de Freitas 45992-255, Brazil; ajsantos.ppgef@uesc.br; 3 Center for Training in Public Policies and Social Technologies, Federal University of Southern Bahia, Itabuna 45600-923, Brazil

**Keywords:** exercise, students, cross-sectional studies

## Abstract

**Highlights:**

**Public health relevance—How does this work relate to a public health issue?**

- It examines racial inequalities in self-rated health among university students, a strategic population group for health promotion policies.
- It analyzes how different patterns of leisure-time physical activity, including the profile of “weekend warriors,” relate to health perception in unequal social contexts.

**Public health significance—Why is this work of significance to public health?**

- It shows that the perceived benefits of physical activity are not homogeneous across racial groups, revealing persistent inequities even in young and educated populations.
- It contributes to the understanding of how social and structural determinants shape self-rated health, broadening the focus beyond individual behaviors.

**Public health implications—What are the key implications or messages for practitioners, policy makers, and/or researchers in public health?**

- Leisure-time physical activity was positively associated with self-rated health among university students, with variations according to intensity, practice patterns, and race/skin color.
- Public health strategies should promote diverse physical activity patterns and consider social and contextual differences to support perceived health in young adult populations.

**Abstract:**

Leisure-time physical activity (LTPA) is associated with positive self-rated health (SRH); however, evidence regarding different practice patterns and potential racial differences among university students remains limited. The objective of this study was to estimate the association between LTPA patterns and positive SRH among university students who entered higher education in 2025 at a public university in Brazil. This cross-sectional study analyzed data from 1143 first-year undergraduates. Positive SRH (defined as reporting “good” or “very good” health) was used as the outcome. LTPA (walking and activities of moderate and vigorous intensity) was classified as inactive, insufficiently active, weekend warrior, or regularly active based on the World Health Organization (WHO) guidelines on physical activity. The analyses were stratified by self-reported race/skin color (White students vs. students from other racial/ethnic groups). Prevalence ratios (PRs) and 95% confidence intervals (95% CIs) were estimated using Poisson regression. The prevalence of positive SRH was 44.6% among White students and 41.1% among other racial/ethnic group students. Among White students, positive SRH was associated with walking performed at weekend warrior (PR = 2.08; 95% CI: 1.33–3.24) and regular levels (PR = 1.51; 95% CI: 1.06–2.14), as well as with vigorous-intensity activity in a weekend warrior pattern. Among other racial/ethnic group students, positive SRH was associated with regular walking (PR = 1.34; 95% CI:1.05–1.71) and with vigorous-intensity activity at both insufficient and regular levels. LTPA was positively associated with SRH, with variations according to intensity, practice patterns, and race/skin color, indicating that benefits are not homogeneous across groups.

## 1. Introduction

Physical activity represents one of the main health-related behaviors [1]. Insufficient levels may contribute to the development of noncommunicable chronic diseases [2]. On the other hand, it is noteworthy that a portion of the population does not adopt this behavior regularly [3]. In this context, it is essential to recognize, within Brazil, that physical activity does not represent a choice but, in many situations, a necessity [4].

Differences in this behavior are linked to social factors, such as racial inequalities, with individuals who self-identify as White being more active in the leisure-time domain [5]. In Brazil [6,7] and other countries, such as the United States of America [8], race/skin color is an important social determinant of health, reflecting structural inequalities that influence access to resources, health behaviors, and health outcomes. These disparities are consistent with broader evidence indicating that social determinants play a central role in shaping health inequities across populations. Therefore, examining potential differences according to race/skin color may contribute to a better understanding of health disparities in this population.

According to the 2022 Brazilian Census, Black and Brown individuals account for 55.5% of the population, 0.6% self-identify as indigenous, and 0.4% as being Asian [9]. The Census also showed that the proportion of Brazilians aged 25 years or older with higher education was greater among White individuals (25.0%) compared with Brown (12.3%) and Black (11.7%) individuals [6].

Active leisure time, which includes the actions of engaging in exercise and other leisure-time physical activities that contribute to the attainment and maintenance of different health indicators, represents an important opportunity to meet physical activity guidelines [10]. Evidence indicates that leisure-time physical activity is consistently associated with better self-rated health [11,12] (which comprises an important health indicator [13]) in different population groups, such as university students [14].

The number of university students in Brazil has increased in recent years [15], and entry into higher education is associated with lower levels of positive self-rated health [16]. The academic environment may impact leisure-time physical activity patterns, such as the need to accumulate practice on a few days per week, commonly referred to as the weekend warrior pattern (people who accumulate the recommended amount of physical activity within one or two days per week, rather than distributing it across multiple days) [17].

The weekend warrior physical activity practice has demonstrated protective effects against all-cause mortality, cardiovascular disease mortality, cancer mortality, and the occurrence of depression [18]. The literature has not identified an association between leisure-time physical activity patterns, such as the weekend warrior pattern, and self-rated health [18], specifically among university students.

Therefore, this study sought to address potential gaps regarding the leisure-time physical activity profile in this population, considering potential social impacts due to ethnic–racial factors and how this behavior affects overall health. The objective of this study was to estimate the association between leisure-time physical activity patterns, including the weekend warrior pattern, and positive self-rated health among university students who entered higher education in 2025 at a public university in Brazil.

## 2. Materials and Methods

The cross-sectional data of this study were derived from the baseline of a longitudinal study conducted with university students entering the first academic semester of 2025 at a public university located in the city of Ilhéus (municipal HDI: 0.690) [19], in the state of Bahia, Brazil (HDI: 0.786) [19], approximately 500 km from the state capital. The target population for entry in the first academic semester comprised 1344 university students.

University students with active enrollment in one of the institution’s on-campus undergraduate programs were included, namely Agronomic Engineering; Geography (Teacher Education); Geography (Bachelor’s degree); Veterinary Medicine; Business Administration; Accounting Sciences; Biology (Teacher Education); Biology (Bachelor’s degree); Biomedicine; Economics; Production and Systems Engineering; Civil Engineering; Chemical Engineering; Mechanical Engineering; Electrical Engineering; Chemistry (Teacher Education); Chemistry (Bachelor’s degree); Physics (Teacher Education); Physics (Bachelor’s degree); Mathematics (Teacher Education); Mathematics (Bachelor’s degree); Computer Science; Pedagogy; Nursing; Medicine; Physical Education (Teacher Education); Legal Sciences; Social Sciences (Teacher Education); History (Teacher Education); Philosophical Sciences; Portuguese and Spanish Language and Literature (Teacher Education); Portuguese and English Language and Literature (Teacher Education); Applied Foreign Languages to International Business; Social Communication; and Psychology.

University students enrolled in distance education programs, those with special enrollment status (individuals holding a higher education degree who were enrolled in undergraduate course subjects), and those admitted to positions reserved for holders of a higher education degree or enrolled through external transfer were excluded. University students under 18 years of age at the time of the first survey were also excluded.

Data collection was conducted by convenience sampling among university students between March and July 2025. Active recruitment of university students took place in the institution’s classrooms, before or after classes on different days of the week, aiming to ensure an appropriate and similar setting for participation in the study.

University students freely self-administered the questionnaire, with assistance from trained administrators to clarify potential doubts. The questionnaire was administered simultaneously to all eligible university students, while ensuring privacy and confidentiality across individual participation. The average completion time was 30 min. Each university student was assigned a numerical identification code.

Information was measured using instruments validated for application in studies with Brazilian university students and young adults [20,21]. Sociodemographic questions and items related to the students’ relationships with the university were incorporated into the questionnaire. The dependent variable of this study was self-rated health. Self-rated health was assessed using the question “In general, how would you rate your current health status?”, with response options of very poor, poor, fair, good, and very good. For the purposes of this study, responses of “good” and “very good” were grouped and defined as positive self-rated health. The reproducibility level of this measure is 83.9% (Kappa: 0.70) [21].

The independent variables in this study were three leisure-time physical activity patterns, including activity types (walking) and intensity levels (moderate and vigorous) (Table 1), assessed using the long version of the International Physical Activity Questionnaire (IPAQ) [20]. Although this instrument allows the evaluation of physical activity across multiple domains (leisure, work, commuting, and household), only the leisure-time domain was used to classify physical activity patterns, while the other domains were included as control variables in the adjusted analyses. Leisure-time physical activity patterns were classified according to weekly duration thresholds consistent with international physical activity recommendations, including the World Health Organization guidelines [22]. Time spent in vigorous-intensity physical activity was multiplied by two to reflect its higher energy expenditure compared to moderate-intensity activity, following the IPAQ scoring protocol described in the original validation study [20]. This approach was investigated in a study conducted with Brazilian university students [23].

The stratification variable of the study was self-reported race/skin color, dichotomized into White and other racial/ethnic groups (Black, Brown, Asian, and Indigenous) [24]. The control variables included gender (male and female), age (18–19 and 20–69 years), study period (daytime and nighttime), class hours per week (dichotomized at the median: up to 24.9 and ≥25 h), marital status (with partner and without partner), and personal income (none and up to one minimum wage (BRL 1518.00) or more). Sleep duration [21] was classified as adequate (7–9 h per day) or inadequate (<7 or >9 h per day), based on recommendations from the National Sleep Foundation [25]. Sitting time [20] was categorized as low/moderate (≤9.27 h per day) or high (>9.27 h per day), based on thresholds previously estimated in a study with Brazilian university students, compared to sedentary behavior measured by accelerometry [26]. Sleep time and sitting time were calculated using a weighted average, and these measurements showed satisfactory levels of reproducibility in Brazilian populations [27,28]. Additionally, physical activity in other domains (work, commuting, and household) was included as a control variable and measured in minutes per week using the IPAQ [20].

The questionnaires were digitized through optical reading using the Remark software (version 11). Descriptive analyses of absolute and relative frequencies, means, and standard deviations were performed. Differences in proportions between race/color skin groups were assessed using the chi-squared test for both the outcome (self-rated health) and the independent variables (leisure-time physical activity patterns). The measure of association used was the prevalence ratio (PR), complemented by 95% confidence intervals (95% CIs), estimated via Poisson regression with robust variance adjustment, in both crude and adjusted analyses. In each model, one domain-specific pattern (walking, moderate-intensity, and vigorous-intensity activities) was treated as the main independent variable, while the remaining leisure-time physical activity patterns were included as covariates, along with other control variables. In the adjusted analysis, all control variables were included together with all independent variables, and the final adjusted model comprised the control variables presenting a Wald test *p*-value > 0.20, removed one at a time. This criterion of remotion did not apply to the remaining leisure-time physical activity patterns. The adopted significance level was 5%.

## 3. Results

### 3.1. Sample Characteristics

Of the number of students entering university in 2025, 1143 undergraduates participated in the study (response rate: 74.9%). Among the 382 non-participants, 22 were excluded at the time of data collection because they reported being under 18 years of age (n = 22). In addition, 37 students declined to participate, and the remaining individuals could not be located. The sample characteristics are presented in Table 2. The majority of participants were of other racial/ethnic groups (Brown: 40.6%; Black: 32.0%; Asian: 0.4%; Indigenous: 1.5%). Both White students and those of other racial/ethnic groups showed a majority of women, aged 18 and 19, without an income and without a partner. Students of other racial/ethnic groups stood out due to having a class load of 25 h/class per week or more and engaging in physical activity at work, during commutes, and at home.

### 3.2. Leisure-Time Physical Activity Patterns

Table 3 presents the prevalence of leisure-time physical activity patterns according to walking, moderate, and vigorous intensities, along with their respective 95% CIs and comparisons between White students and those of other racial/ethnic groups.

### 3.3. Prevalence of Positive Self-Rated Health

The prevalence of positive self-rated health was 44.6% (95% CI: 38.9–50.4) among White undergraduate students and 41.1% (95% CI: 37.8–44.5) among other racial/ethnic groups, with no statistically significant difference between groups (χ^2^ = 1.06; *p* = 0.303).

### 3.4. Associations Between Leisure-Time Physical Activity and Positive Self-Rated Health Among White and Other Racial/Ethnic Group Students

In relation to the White university students, in the crude analyses (Table 4), higher prevalences of positive self-rated health were observed in leisure-time physical activity across all modalities. For walking, insufficiently active, weekend warrior, and regularly active students showed higher prevalences compared with inactive peers. Similar associations were found for moderate and vigorous intensities, particularly among weekend warriors and regularly active undergraduates, whereas insufficiently active students showed no consistent associations. After adjustment (Table 4), the associations were attenuated, and walking remained positively associated with the outcome among weekend warriors and regularly active students. For moderate intensity, no significant associations persisted after adjustment. For vigorous intensity, only the weekend warrior pattern remained independently associated with a higher prevalence of positive self-rated health.

In relation to university students in the other racial/ethnic groups, in the crude analyses, higher prevalences of positive self-rated health were observed in leisure-time physical activity across all modalities (Table 4). For walking, insufficiently active and regularly active students showed higher prevalences compared with inactive peers, whereas no significant association was observed among weekend warriors. Similar positive associations were identified for moderate and vigorous intensities, particularly among insufficiently active and regularly active students. After adjustment, most associations were attenuated (Table 4). Walking remained positively associated with the outcome only among regularly active university students. For moderate intensity, no significant associations persisted after adjustment. For vigorous intensity, insufficiently active and regularly active students remained independently associated with a higher prevalence of positive self-rated health, while no significant associations were observed for the weekend warrior pattern.

## 4. Discussion

The main findings of this study indicated that leisure-time physical activity was associated with higher prevalences of positive self-rated health among Brazilian university students entering higher education, with relevant differences according to the practice pattern, activity intensity, and self-reported race/skin color. Overall, more consistent practice patterns—especially regular physical activity and, in some cases, the weekend warrior pattern—were associated with positive health perception.

Among university students who self-identified as White, leisure-time walking remained associated with positive self-rated health for both the weekend warrior pattern (PR: 2.08; 95% CI: 1.33–3.24) and regular practice (PR: 1.51; 95% CI: 1.06–2.14). This result corroborates findings from another study of Brazilian adults, in which leisure-time walking, whether at intermediate levels (150 to 300 min per week) or high levels (300 min or more per week), was directly associated with positive self-rated health [29]. This finding reinforces the relevance of walking as an accessible and potentially health-promoting modality [30], especially in contexts in which weekly regularity may be limited by academic and personal demands, such as among university students.

In the same group of university students, within the vigorous-intensity physical activity domain, only the weekend warrior pattern remained independently associated with better self-rated health (PR: 1.49; 95% CI: 1.08–2.06), suggesting that accumulating volumes of physical activity at this intensity on a few days may enable a better perception of health. This result aligns with other studies conducted with adolescents from different countries [31] and Spanish adults [32], which reported an association between vigorous-intensity leisure-time practice and positive perception. It is plausible that university students engaged in higher-intensity activities, even on a few days, may present greater self-efficacy and physical fitness levels, which favor more positive health perception.

Conversely, among university students of other racial/ethnic groups, a distinct pattern was observed. Walking remained positively associated with positive self-rated health only among those who were regularly active (PR: 1.34; 95% CI: 1.05–1.71). Regarding vigorous physical activity, both insufficiently active and regularly active students presented higher prevalences of positive health perception. These results suggest that, for other racial/ethnic group students, the frequency of practice, whether meeting guidelines or not, may play a relevant role in health perception.

Differences between groups according to race/skin color may be related to social and contextual inequalities that influence both access to and the experience of active leisure, as White Brazilians tend to be more active during leisure time than their self-identified Black or Brown peers [5], as well as to risks related to different health indicators, such as depression, which shows lower levels of occurrence among White individuals under conditions of insufficient practice, the weekend warrior pattern, and regular practice [33]. However, although understanding the role of physical activity in the Brazilian context is important [4], it is necessary to highlight the need for further studies, especially longitudinal investigations of these groups over time, given the potential inconclusiveness regarding differences in leisure-time physical activity patterns according to race/skin color [34].

The findings also indicated that, after adjustments, associations involving moderate-intensity physical activity did not remain significant in either group. This result may reflect the heterogeneity of practice at this intensity [35]. On the other hand, it should be considered that physical activity at levels below a vigorous intensity can be fundamental for the occurrence of positive health perception [36]. Furthermore, lower risks of all-cause mortality may be achieved through practice exclusively at a moderate intensity or exclusively through vigorous-intensity physical activity [37].

In this study, the prevalence of positive self-rated health was slightly higher among White students compared to other racial/ethnic group students; however, this difference was not statistically significant. These prevalences were lower when compared with systematized findings on the topic among university students [14] and consistent with previous studies demonstrating that racial differences in self-rated health reflect persistent structural inequalities [38].

Population-based studies indicate that individuals of other racial/ethnic groups are more likely to report fair or poor health compared with White individuals, even after adjustment for socioeconomic factors [39], suggesting that social determinants, experiences of discrimination, and unequal access to health resources directly influence subjective health perception. In the university context, one study suggests that other racial/ethnic group students tend to experience greater exposure to psychosocial stressors, including perceived discrimination, which may negatively impact well-being and, consequently, positive self-rated health [40].

The positive self-rated health identified among university students may be associated with specific characteristics of the sample and the institutional context, such as socioeconomic conditions and available social support, which were statistically similar among the groups investigated in this study. Therefore, in this study, positive self-rated health may reflect regional similarities between the groups and, above all, the influence of contextual factors such as academic stress and social vulnerabilities, as previously mentioned. Thus, the findings suggest that self-rated health among university students should be interpreted in light of the social contexts in which these young individuals are embedded.

This study expands the literature by investigating, for the first time among Brazilian university students, the association between different leisure-time physical activity patterns, including the weekend warrior pattern, and self-rated health, considering aspects inherent to inequalities present in the Brazilian population due to skin color [34]. Although not the primary focus of this study, future research should further explore gender differences in physical activity patterns and self-rated health, as these factors may interact with social and contextual determinants. Previous evidence indicates that men and women may differ in both physical activity patterns and self-rated health [3,11].

Limitations should be acknowledged, such as the use of self-reported information regarding physical activity practices measured through the long version of the IPAQ, which may suggest possible recall bias; nevertheless, the instrument demonstrates satisfactory levels of validity and reproducibility [20]. Furthermore, considering the structure of the physical activity questions, it was not possible to investigate total leisure-time physical activity patterns (sum of walking and moderate- and vigorous-intensity activities). Although the results refer to a single public university, it is important to highlight that the gender and age profile corroborates data from the 2024 Higher Education Census from Brazil [15].

## 5. Conclusions

It is concluded that leisure-time physical activity is associated with higher prevalences of positive self-rated health among university students, with variations according to the practice pattern, activity intensity, and self-reported race/skin color. Among White university students, positive associations were observed for walking and for vigorous-intensity leisure-time physical activity under the weekend warrior pattern, whereas, among university students from other racial/ethnic groups, regularity of practice was more consistently associated with positive health. These results indicate that, although the weekend warrior pattern may represent a viable strategy to promote positive health perception in certain groups, its effects are not homogeneous among university students entering higher education. Therefore, interventions and policies promoting physical activity within the university context should consider social and racial inequalities, as well as the value of multiple forms of engagement in active leisure, respecting the conditions and opportunities available to different population groups.

## Figures and Tables

**Table 1:** Description of the classification criteria for leisure-time physical activity practice.

Pattern	Categories	Definition
Walking	Inactive	No days per week
	Insufficiently active	1 or more days per week and total practice time up to 149 min
	Weekend warrior	1 to 2 days per week and total practice time of 150 min or more
	Regularly active	3 or more days per week and total practice time of 150 min or more
Moderate intensity	Inactive	No days per week
	Insufficiently active	1 or more days per week and total practice time up to 149 min
	Weekend warrior	1 to 2 days per week and total practice time of 150 min or more
	Regularly active	3 or more days per week and total practice time of 150 min or more
Vigorous intensity	Inactive	No days per week
	Insufficiently active	1 or more days per week and total practice time up to 149 min
	Weekend warrior	1 to 2 days per week and total practice time of 75 min or more
	Regularly active	3 or more days per week and total practice time of 75 min or more

**Table 2:** Descriptive characteristics of college students. Brazil, 2025.

Variables	White (n: 287)		Other Racial/Ethnic Groups (n: 839)	
Categorical variables	n	%	n	%
Gender				
Male	121	43.5	345	41.8
Female	157	56.5	481	58.2
Age, years				
18 and 19	187	67.5	548	66.7
20 to 69	90	32.5	273	33.3
Period of study				
Day	217	75.6	575	68.5
Night	70	24.4	264	31.5
Class hours per week				
Up to 24.9	115	40.1	426	50.8
25 or more	172	59.9	413	49.2
Marital status				
With partner	209	73.1	598	71.6
Without partner	77	26.9	237	28.4
Personal income				
I don’t have	200	71.2	576	69.5
Up to 1 minimum wage or more	81	28.8	253	30.5
Sleep time				
Adequate	157	54.9	396	47.9
Inadequate	129	45.1	431	52.1
Sitting time				
Lower/Moderate	188	66.9	551	68.6
Higher	93	33.1	252	31.4
**Numerical variables**	**n**	**Mean (SD)**	**n**	**Mean (SD)**
Physical activity at work (min/week)	266	118.75 (279.06)	772	172.88 (335.96)
Commuting (min/week)	286	230.17 (269.29)	829	274.07 (304.36)
Physical activity at home (min/week)	287	322.99 (308.57)	836	436.50 (360.47)

SD: standard deviation; min: minutes. Missing data were present for some variables; therefore, the total number of observations may vary across categories and may not equal the overall sample size.

**Table 3:** Prevalence of leisure-time physical activity patterns according to walking, moderate, and vigorous intensities among university students by race/skin color. Brazil, 2025.

Modality of Leisure-Time Physical Activity	n	White, % (95% CI)	n	Other Racial/Ethnic Groups, % (95% CI)	*p*-Value
Walking					0.267
Inactive	174	60.8 (55.0–66.3)	524	62.8 (59.5–66.0)	
Insufficiently active	68	23.8 (19.2–29.1)	157	18.8 (16.3–21.6)	
Weekend warrior	5	1.7 (0.7–4.1)	21	2.5 (1.6–3.8)	
Regular active	39	13.6 (10.1–18.1)	132	15.8 (13.5–18.5)	
Moderate					0.358
Inactive	178	62.2 (56.4–67.7)	554	66.3 (63.0–69.5)	
Insufficiently active	30	10.5 (7.4–14.6)	96	11.5 (9.5–13.8)	
Weekend warrior	25	8.7 (5.9–12.6)	63	7.5 (5.9–9.5)	
Regular active	53	18.5 (14.4–23.5)	122	14.6 (12.4–17.2)	
Vigorous					0.984
Inactive	176	61.5 (55.7–67.0)	524	62.8 (59.5–66.0)	
Insufficiently active	9	3.1 (1.6–5.9)	25	3.0 (2.0–4.4)	
Weekend warrior	30	10.5 (7.4–14.6)	86	10.3 (8.4–12.6)	
Regular active	71	24.8 (20.1–30.2)	199	23.9 (21.1–26.9)	

Values are expressed as prevalence (%); 95% CI: 95% confidence interval. Differences between groups were tested using the chi-squared test. Missing data were present for some variables; therefore, the total number of observations may vary across categories and may not equal the overall sample size.

**Table 4:** Associations between leisure-time physical activity modalities according to walking practices and moderate and vigorous intensities in White and other racial/ethnic groups among university students. Prevalence ratios estimated via Poisson regression. Brazil, 2025.

Modality of Leisure-Time Physical Activity	White University Students				Other Racial/Ethnic Group University Students			
	n	%	PR_Crude_ (95% CI)	PR_Adjusted *_ (95% CI)	n	%	PR_Crude_ (95% CI)	PR_Adjusted **_ (95% CI)
Walking								
Inactive	63	36.2	1.00	1.00	189	36.1	1.00	1.00
Insufficiently active	35	51.5	1.42 (1.05–1.93)	1.22 (0.90–1.65)	71	45.2	1.66 (1.39–1.99)	1.08 (0.86–1.36)
Weekend warrior	5	100.0	2.76 (2.27–3.36)	2.08 (1.33–3.24)	5	23.8	0.66 (0.30–1.43)	0.80 (0.41–1.56)
Regular active	25	64.1	1.77 (1.30–2.41)	1.51 (1.06–2.14)	79	59.8	1.66 (1.39–1.99)	1.34 (1.05–1.71)
Moderate								
Inactive	66	37.1	1.00	1.00	192	34.7	1.00	1.00
Insufficiently active	14	46.7	1.26 (0.82–1.93)	0.97 (0.65–1.45)	53	55.2	1.59 (1.29–1.97)	1.17 (0.91–1.51)
Weekend warrior	15	60.0	1.62 (1.11–2.35)	1.24 (0.87–1.78)	32	50.8	1.47 (1.12–1.92)	1.12 (0.84–1.49)
Regular active	33	62.3	1.68 (1.26–2.23)	1.21 (0.89–1.65)	67	54.9	1.58 (1.30–1.93)	1.04 (0.80–1.35)
Vigorous								
Inactive	64	36.4	1.00	1.00	179	34.2	1.00	1.00
Insufficiently active	2	22.2	0.61 (0.18–2.11)	0.72 (0.23–2.27)	13	52.0	1.52 (1.03–2.26)	1.56 (1.07–2.28)
Weekend warrior	19	63.3	1.74 (1.25–2.43)	1.49 (1.08–2.06)	35	40.7	1.19 (0.90–1.58)	1.06 (0.78–1.43)
Regular active	43	60.6	1.66 (1.27–2.18)	1.26 (0.94–1.70)	117	58.8	1.72 (1.46–2.03)	1.35 (1.09–1.67)

PR: prevalence ratio; 95% CI: 95% confidence interval; * Adjusted: gender, period of study, sitting time, modality of leisure-time physical activity, physical activity at home; ** Adjusted: gender, age in years, period of study, marital status, sleep time, modality of leisure-time physical activity, commuting, physical activity at home and work.

## Data Availability

Dataset available on request from the authors. The data are not publicly available due to privacy and ethical restrictions.

## Abbreviations

IPAQ	International Physical Activity Questionnaire
HDI	Human Development Index
